# Editorial: Redox control of plant metabolism and biofuel production

**DOI:** 10.3389/fpls.2023.1244229

**Published:** 2023-07-24

**Authors:** Ramachandran Sivaramakrishnan, Daniel A. Gideon, S Prashant Jeevan Kumar, Aran Incharoensakdi

**Affiliations:** ^1^ Laboratory of Cyanobacterial Biotechnology, Department of Biochemistry, Faculty of Science, Chulalongkorn University, Bangkok, Thailand; ^2^ Department of Biotechnology and Bioinformatics, Bishop Heber College (Autonomous), Affiliated to Bharathidasan University, Tiruchirappalli, Tamil Nadu, India; ^3^ Department of Biochemistry, St. Joseph’s University, Bengaluru, Karnataka, India; ^4^ Indian Council of Agricultural Research (ICAR)-Directorate of Floricultural Research, Pune, Maharashtra, India; ^5^ Academy of Science, Royal Society of Thailand, Bangkok, Thailand

**Keywords:** redox, microalgae (blue green algae, plant metabolism, ROS, antioxidative

During the ongoing metabolism of plants the generation of reactive oxygen species (ROS) is not uncommon. Plants normally have a redox signaling system to minimize ROS and to maintain the balance of redox metabolism. Excessive production of ROS occurs when plants are under environmental stress conditions such as drought, high salinity, heavy metals contamination, pathogens attack, etc. In general, ROS can be scavenged by various antioxidative defense systems in plants.

The first article in this Research Topic explores the molecular mechanism of citrus fruit resistance against *Penicillium digitatum* infection (Wang et al.). WRKY is a transcription factor with a role in the regulation of biotic stress. The authors found the upregulation of *CsWRKY25* gene in *P. digitatum* infected citrus peel. The *Agrobacterium*-mediated transient overexpression of *CsWRKY25* led to increased resistance to *P. digitatum* in citrus mediated by the accumulation of reactive oxygen species with subsequent activation of the antioxidant system.

Another *study* by Shahid et al. used plant growth promoting rhizobacterium (PGPR) strain to enhance the salt tolerance in radish to increase the radish production. The isolated strain efficiently produced the essential plant growth promoting compounds like indole-3-acetic acid (IAA), siderophore (iron-chelating compounds), ammonia, and 1-amino cyclopropane 1-carboxylate deaminase. All these compounds were elevated during saline stress. PGPR enhanced the efficiency of radish and reduced the toxicity of NaCl. The authors concluded that the PGPR strain could enhance the radish production under salinity soil conditions.

The tomato plant grown under lead (Pb) contaminated soil was investigated by Ma et al. They observed improved properties of tomato by applying syringic acid exogenously. Increasing Pb level in the soil decreased the shoot length, dry weight of shoot and root, reduced the photosynthetic pigments such as chlorophylls and carotenoids content, reduced the photosynthesis efficiencies, sugar content, transpiration rate and non-reducing sugar contents by increasing ROS molecules which disturbs the redox equilibrium and affect the metabolic activities of cells. Pb toxicity resulted in the increase of malondialdehyde, hydrogen peroxide and electron outflow causing the oxidative stress in shoots and roots. The exogenous supplementation of syringic acid reduced Pb toxicity in tomato plants by decreasing the ROS production, reducing Pb contents in plant organs, and maintaining essential minerals in plants.

The enhancement of the odor characteristics of Wuyi rock tea was studied by Jia et al. using a traditional deep fermentation method. They were able to identify 17 different characteristic compounds responsible for the aroma of the Wuyi rock tea. Dihydromyrcenol is an important compound responsible for the floral aroma, whereas six terpenoids [(E)-sabinene, isopinocarveol, aristolochene, deltacadinene, myrtenol, (E)-germacrene D] produced during the fermentation give the woody aroma, and six esters give the fruity odor mainly from the (Z)-3-hexen-1-yl butyrate, (E)-3-hexen-1-yl butyrate, 5-hexenyl butyrate, hexyl butyrate, hexyl 2-methyl butyrate, and (Z)-3-hexen-1-yl 2-methyl butyrate. The authors concluded that the traditional deep fermentation based volatile metabolites could enhance the initial processing of Wuyi rock tea.

The last review article on the advances of understanding the ROS and its regulation in seed dormancy, germination and deterioration in crops was presented by Li et al. Scavenging mechanism of ROS in seeds and its regulation in germination of seeds and their dormancy were mainly discussed. The regulation of ROS and its maintenance in seeds is necessary to enhance the germination and its potential, and to avoid seed deterioration. The review further discussed the advancement of ROS regulation in hydrated and dry seed crops during homeostasis. The authors also discussed the relationship between plant hormones such as gibberellic and abscisic acid, and ROS regulation and its influence during germination of crops and seed dormancy. Mechanisms involved in the seed deterioration and its repair system during the regulation of ROS were also explored.

Redox mechanisms are involved in all biochemical reactions in plants. Therefore, the exact mechanisms which control plant physiology must be explored. Various proteins, chemicals and redox-mediated metabolites are involved in the maintenance of redox homeostasis under normal as well as challenged environmental conditions. With respect to the fluctuations of the environmental conditions, the current understanding of cellular redox control may be crucial in terms of the responses against ROS. An intricate network of chemo-enzymatic redox system controls all biochemical processes and metabolism in plant cells. The plant growth is mainly controlled by redox-dependent mechanisms and interactions of ROS, phytohormones such as ethylene, cytokinin and auxin, and antioxidants. The changes in redox environment can induce various physiological regulatory networks such as reactive nitrogen species, ROS and antioxidants. Further advances in the redox mechanism-based research may give the chance to researchers to refresh their understanding of the redox based plant functions.

In summary, all five articles in this Research Topic with discussion on the redox mechanisms will benefit researchers working in this field to improve the understanding of redox based plant regulations with respect to the rapid changes in extreme environmental conditions. Last but not least, it was unfortunate that there was no submission to this Research Topic on the aspect of redox control with respect to biofuel production.

## Author contributions

RS: Conceptualization, writing- original draft, Writing - review & editing. DG: Writing - review & editing. SK: Writing - review & editing. AI: Supervision, Project administration, Writing - review & editing. All authors contributed to the article and approved the submitted version

